# Preschoolers’ early gaze exploration in virtual preschool classrooms: a transition-based eye-tracking analysis of window views and room size

**DOI:** 10.3389/fpsyg.2026.1813117

**Published:** 2026-05-12

**Authors:** Kijoo Cha, Hanseob Kim, Dong-Hyeon Kim

**Affiliations:** 1 Graduate School of Education, Ewha Womans University, Seoul, Republic of Korea; 2 Department of XR for National Defense, Konyang University, Nonsan-si, Republic of Korea; 3 Department of IT Convergence Engineering, Gachon University, Seongnam-si, Republic of Korea

**Keywords:** classroom size, eye-tracking, gaze transitions, preschool classroom, scanpath, spatial exploration, virtual reality, window view

## Abstract

Preschool age is a developmental period in which children are learning to orient in unfamiliar spaces while still relying strongly on visible landmarks and other salient environmental cues, yet little is known about how classroom design shapes this initial orienting process. To address this gap, this study examined how preschoolers (ages 5–6) visually explore immersive virtual preschool classrooms during the first 5 min of exposure using head-mounted virtual reality (VR) with integrated eye tracking. Two architectural manipulations were tested in separate between-subject comparisons: window-view content (city vs. nature) and classroom size (large vs. small). Gaze was mapped to classroom activity areas (areas of interest; AOIs) and analyzed using (a) proportional dwell time and (b) transition-based metrics derived from AOI sequences (e.g., transition entropy, return-to-anchor dynamics), followed by unsupervised clustering of scanpath patterns. In the window-view comparison, children in the nature-view classroom allocated more gaze to window-related AOIs (i.e., central and side windows). Two exploration patterns emerged: a return-anchored pattern characterized by frequent returns to a window reference, and an expansion-oriented pattern marked by broader outward exploration; cluster membership was strongly associated with view condition. In the classroom-size comparison, large and small rooms showed distinct AOI prioritization and process-level exploration profiles, with larger rooms eliciting more rapid and/or diverse scanning and smaller rooms eliciting more repetitive or focal viewing. Overall, the results suggest that window-view content and room scale systematically reorganize the temporal structure and spatial distribution of preschoolers’ early visual orientation in immersive learning environments, providing initial evidence for developmentally informed classroom design.

## 1 Introduction

When individuals first encounter an unfamiliar interior environment, they typically engage in rapid visual scanning to sample spatial layout, salient openings, and potential reference cues for orientation. Evidence for this process comes from both non-VR and VR-based studies of built-space experience. In non-VR settings, gaze has been examined in physical installation and museum environments, where observers initially sample salient features and spatial structure while viewing unfamiliar interiors (Gulhan et al., 2021; Mandolesi et al., 2022). Related VR work has shown that visual attention in interior settings is likewise shaped by room cues such as window views, perceived spaciousness, and patient-room composition (Sathyanarayanan et al., 2024, 2025; Tural and Tural, 2024). Although these studies differ in context, they converge on a common point: during initial exposure to an unfamiliar space, gaze is organized not randomly but around visually salient and spatially informative cues that can anchor subsequent appraisal and exploration. Early sampling behavior is therefore relevant not only to perceptual selection but also to the establishment of reference information for orientation and wayfinding in built spaces.

Among built spaces, this study particularly focuses on classrooms for preschool-aged children. Early childhood is a developmental period in which children are learning to orient in unfamiliar spaces while still relying strongly on visible landmarks and other salient environmental cues (Jung and Dilks, 2025; Lingwood et al., 2015; Nys et al., 2015; Siegel and White, 1975; van Hoogmoed et al., 2022). Yet little is known about how physical characteristics of learning environments shape this initial orienting process among young children, especially as examined through eye-tracking technology. Accordingly, this study examines how variations in VR preschool classroom design shape the early visual exploration of 5–6-year-old children.

Early gaze behavior during spatial exploration can be interpreted in light of work on environmental legibility and wayfinding, which emphasizes that navigation depends not only on individual ability but also on how readily spatial structure and reference information can be extracted for orientation and decision-making (Passini, 1984; Weisman, 1981; Jamshidi et al., 2020). A central mechanism in this literature is landmark use: visually and cognitively salient environmental features support self-localization and route choice, particularly when they are referenced in route directions and positioned at decision points (Nothegger et al., 2004; Yesiltepe et al., 2021). In complex interior settings, stable distal cues that remain visible across viewpoints—together with floor-plan complexity and building configuration—have been shown to shape wayfinding performance and early orientation (Hölscher et al., 2006; O’Neill, 1991).

This framework is developmentally relevant because children do not use environmental information in the same way as adults. Classical developmental theory proposes that large-scale spatial representations develop from landmark knowledge to route knowledge and, later, to more integrated survey knowledge (Siegel and White, 1975). Empirical work in virtual environments is broadly consistent with this progression: children aged 6–10 show age-related increases in the quantity and precision of remembered landmarks, in the selection of functionally useful landmarks, and in the ability to bind landmarks to directional information (Nys et al., 2015). Similarly, landmark-based navigation improves across ages 5–10, with age-related gains in how flexibly children use different landmark types to support navigation (van Hoogmoed et al., 2022). Research further indicates that children do not treat all cues as equally informative: typically developing children aged 6–9 show better recall for junction-adjacent landmarks than for landmarks located away from decision points in a virtual environment (Farran et al., 2012), and even 5-year-olds can improve route learning when their attention is explicitly directed toward landmarks during initial exposure (Lingwood et al., 2015). Recent neural evidence further indicates that by age 5 children already encode navigationally relevant location information in a large-scale virtual environment, suggesting that map-relevant location coding emerges early but continues to undergo functional refinement (Jung and Dilks, 2025). Taken together, these findings suggest that during initial exposure to a new classroom scene, preschoolers may be especially sensitive to visible anchors and distal openings, yet exhibit less stable or less efficient scanning than older children and adults.

Gaze behavior during spatial exploration may also be shaped—often implicitly—by visually salient elements within the environment (e.g., color, contrast, distinctive objects), resulting in shifts in attention (Cho and Suh, 2020; Sheng et al., 2025). In adult or college-student samples, prior eye-tracking studies have reported that variations in window size and access to views, materiality, and overall spatial composition can systematically shape where people look and how they appraise interiors (Amundadottir et al., 2017; Mandolesi et al., 2022; Song et al., 2016; Tural and Tural, 2024). For example, Tural and Tural (2024) showed that larger windows in VR interiors increased the frequency with which college students directed their gaze toward outdoor views. Related human-centric daylight modeling work has likewise suggested that daylight distribution and view direction can reorganize predicted gaze-responsive zones across immersive interior scenes (Amundadottir et al., 2017). Together, these findings imply that salient interior features and distal openings (e.g., windows) may jointly shape not only attentional allocation but also the reference structures—that is, potential orientation anchors—available during early orientation. Beyond such feature-level differences, the overall scale and openness of a space may further constrain how exploration unfolds. Yet direct manipulations of these properties remain comparatively rare in eye-tracking research, and still rely predominantly on adult participants (Gao et al., 2025; Kim et al., 2022; Rusnak et al., 2019).

Comparable investigations in preschool-aged children remain scarce. Although eye-tracking studies involving school-age and preschool children have steadily increased, much of this work has focused on developmental and educational psychology contexts—such as reading, language processing, socio-emotional understanding, and spatial reasoning (e.g., Bolden et al., 2015; Cavadini et al., 2024; Hsieh, 2025; Miller et al., 2020; Neumann, 2013; Takacs and Bus, 2018; Verdine et al., 2017)—rather than on real or realistically simulated spatial environments. Child–environment applications remain relatively limited, encompassing studies analyzing visual exploration in streetscapes or examining how window views and environmental elements in VR pediatric hospital rooms relate to visual engagement and emotional responses (e.g., Sathyanarayanan et al., 2024, 2025; Sheng et al., 2025). To date, however, no study has systematically classified preschoolers’ initial exploration patterns in a classroom setting during the earliest phase of visual exposure, nor examined how specific spatial conditions organize those patterns from a transition-based perspective.

Accordingly, the present study analyzed eye-tracking data from preschoolers aged 5–6 during the first 5 min (0–300 s) of seated exposure to a VR preschool classroom. We examined not only (1) how children distributed their attention across classroom play areas (AOI-level gaze proportions), but also (2) how gaze transitions among areas were organized over time (transition-based metrics and clustering). Specifically, we investigated two spatial design conditions: window-view content (city vs. nature) and classroom size (large vs. small). These manipulations allowed us to examine how a salient visual attractor (window view) and a change in spatial scale (classroom size) shape preschoolers’ early exploration dynamics. In addition, we explored whether individual characteristics (sex, age in months, and enrollment duration) were associated with cluster-based exploration profiles within each condition. Given the limited existing research on preschoolers’ visual exploration of learning spaces, these findings provide foundational evidence for developmentally informed classroom design and for future work on early orientation dynamics in learning environments.

### 1.1 Research questions

To examine how preschoolers visually explore a classroom—a spatial context for which they possess an established schema and prior familiarity—during the first 5 min of seated exposure, the present study addresses the following research questions:

Window-view condition

RQ1. How does early classroom exploration differ by window-view condition (city vs. nature) in transition-based clusters?
RQ2. Do child characteristics predict transition-based cluster membership within each window-view condition?

Classroom-size condition

RQ3. How does early classroom exploration differ by classroom size (large vs. small) in process-level exploration clusters?
RQ4. Do child characteristics predict process-level exploration cluster membership within each classroom-size condition?

## 2 Materials and methods

Before describing the current procedures, it is important to note that the dataset analyzed here was not originally collected with the primary aim of examining preschoolers’ eye-tracking responses or scanpath dynamics in relation to manipulated classroom design features. Rather, the VR classroom sessions and associated data were obtained as part of a broader VR classroom project designed to examine how classroom size and window view relate to preschoolers’ cognitive performance and physiological responses. Two prior publications derived from that project addressed research questions and outcomes different from those of the present manuscript: one examined the effects of classroom size and window view on preschoolers’ executive functions and physiological responses (Cha, 2023a), whereas the other examined whether baseline cortisol and negative emotionality moderated those executive-function effects (Cha, 2023b). Neither prior paper reported eye-tracking measures or gaze-based analyses. In contrast, the present manuscript is a distinct secondary analysis focusing specifically on preschoolers’ early visual exploration during initial exposure to a VR classroom. It analyzes gaze at the level of functional areas of interest (AOIs) and characterizes scanpath dynamics using transition-based metrics, including transition entropy and return-to-anchor behavior, followed by unsupervised clustering to identify exploration profiles. These eye-tracking–based, transition-focused analyses and the resulting cluster-based typologies have not been reported in prior publications.

### 2.1 Participants

A total of 144 preschoolers (5–6 years old) residing in the Seoul metropolitan area of South Korea were recruited through online parenting communities widely used by caregivers of preschool-aged children. Children were randomly assigned to one of four VR classroom environments, which were analyzed as two separate between-subject contrasts: (a) a window-view comparison (city- vs. nature-view) and (b) a classroom-size comparison (large- vs. small). One child, originally assigned to the classroom-size condition, was excluded from analysis due to a VR headset recording failure, yielding a final analytic sample of *N* = 143 (61–85 months; *M* = 74.65, SD = 4.84, 73 boys and 70 girls) with 74 children for a window-view comparison (city = 36; nature = 38) and 69 children for a classroom-size comparison (large = 36; small = 33). All participants completed the VR task in a first-time exposure context, viewing the specific virtual classroom environment without prior exposure to the scene.

### 2.2 Procedures

#### 2.2.1 VR Equipment

The virtual preschool classrooms were developed in Unity (version 2020.3.4f1) and implemented using the WorldViz Vizard VR toolkit. Scenes were rendered on a desktop computer and presented through an HTC Vive Pro Eye head-mounted display (HMD). The HMD provides stereoscopic viewing with a 110° horizontal field of view, a total resolution of 2,880 × 1,600 pixels (1,440 × 1,600 per eye), and a 90 Hz refresh rate. Head tracking was achieved via the HMD’s internal sensors (gyroscope, accelerometer, magnetometer) in combination with the external Lighthouse tracking system. Two Lighthouse base stations were positioned at opposite corners of a 2.3 m × 2.3 m tracking area to continuously update the virtual scene according to the participant’s head position and orientation. The integrated Tobii eye tracker in the Vive Pro Eye provides gaze estimates at a nominal sampling rate of 120 Hz, with an estimated spatial accuracy of approximately 0.5°–1.1° (manufacturer specifications). For the present study, gaze and head-pose data were recorded via the SRanipal SDK, including head position (x, y, z), head rotation (quaternion x, y, z, w), and gaze direction vectors (x, y, z). To ensure temporal synchronization between gaze signals and Unity’s fixed update cycle, data were logged at 20-ms intervals, yielding an effective sampling rate of 50 Hz for analysis. Streamlining the data storage to this 50 Hz rate was implemented as a practical choice to synchronize the eye tracking data with the participant behavior data, while maintaining system efficiency and stability throughout the experiment.

#### 2.2.2 VR classroom conditions

**FIGURE 1:**
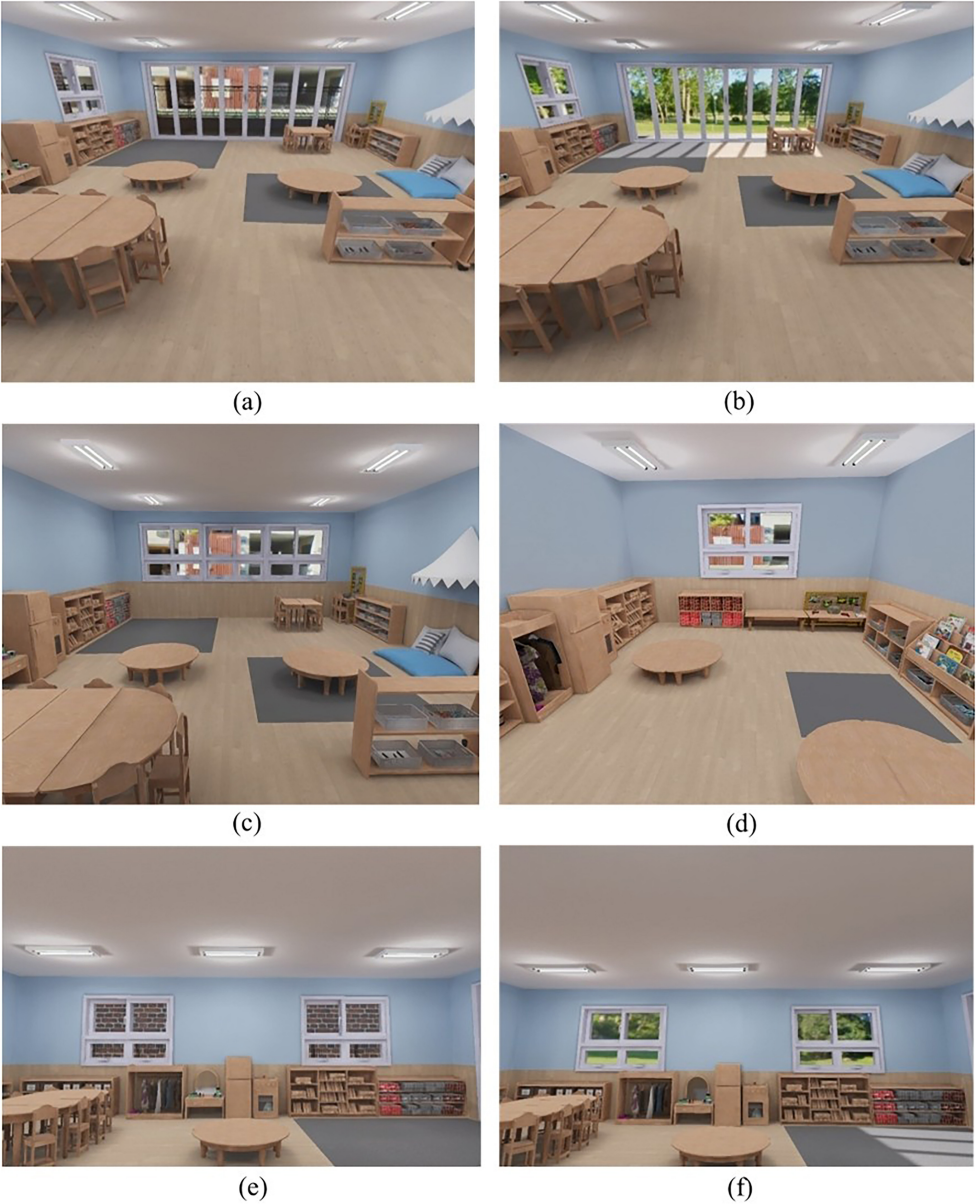
VR preschool classroom conditions and window configurations: **(a)** City view; **(b)** nature view; **(c)** large classroom; **(d)** small classroom; **(e)** small window view in the city-view condition; **(f)** small window view in the nature-view condition. Reproduced from Cha (2023a), licensed under the Creative Commons Attribution 4.0 International License (CC BY 4.0).

Four immersive classroom environments were constructed to represent two manipulated design dimensions: window-view content and classroom size. For the window-view contrast, two classrooms were identical in interior layout but differed in the outdoor scene visible through the windows: a city-view scene (e.g., concrete buildings and streets) versus a nature-view scene (e.g., grass fields and trees) (Figures 1a,b). For the classroom-size contrast, two classrooms differed primarily in overall scale: a large classroom versus a small classroom (Figures 1c,d).

The large classroom dimensions were 2.6 m (H) × 8.9 m (W) × 7.0 m (D). Under the assumption of a class size of 20, this corresponds to approximately 3.1 m^2^ per child, slightly exceeding the OECD average minimum space-per-child guideline for kindergarten/preschool settings (2.9 m^2^) (OECD, 2013). The small classroom measured 2.6 m (H) × 5.0 m (W) × 4.0 m (D), corresponding to approximately 1.0 m^2^ per child (assuming a class size of 20), or roughly one-third the area of the large classroom.

**FIGURE 2:**
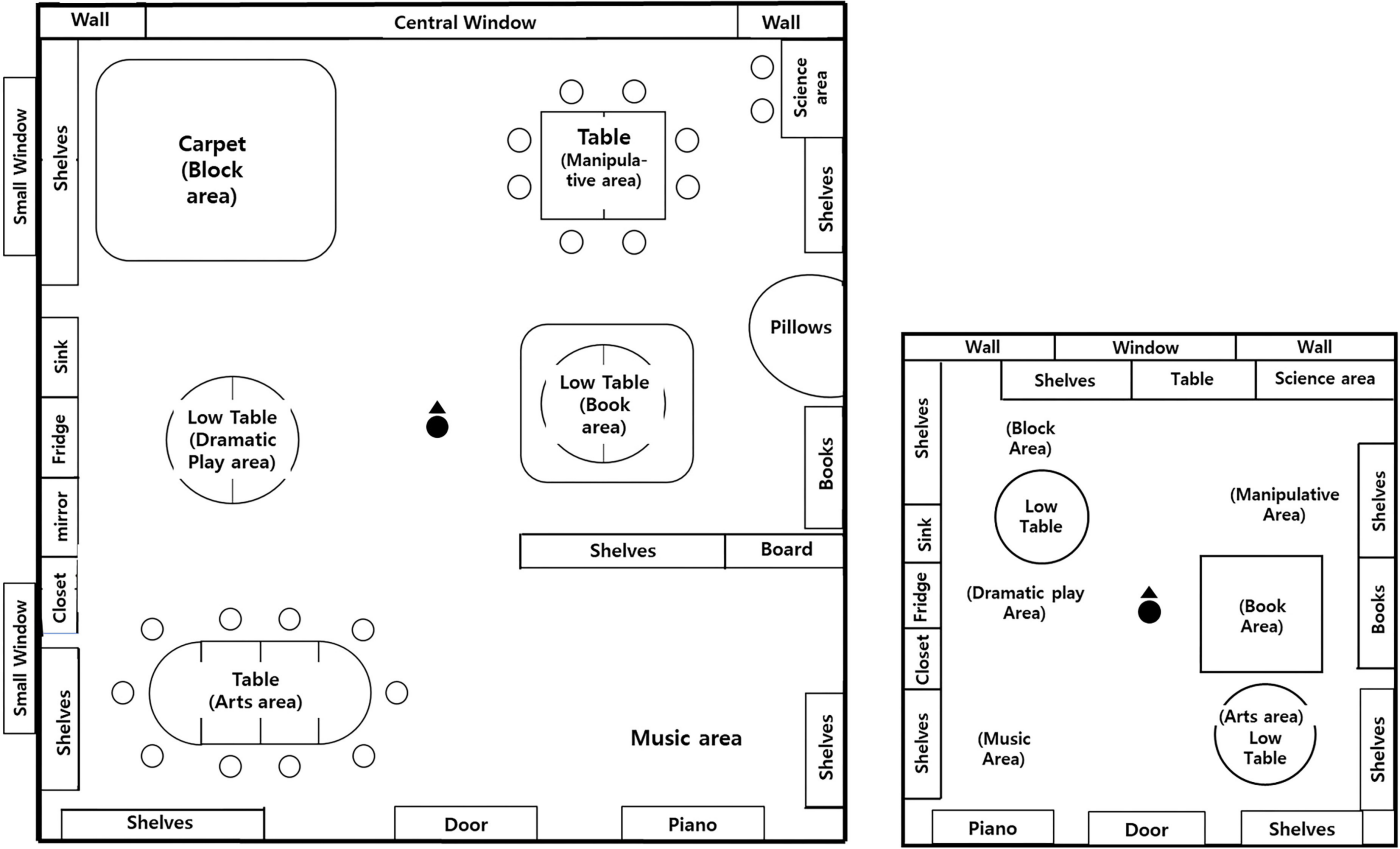
Floor plans and AOI layout for each VR condition: left panel, city-view and nature-view conditions, also used for the large-classroom condition; right panel, small-classroom condition. Source: Supplementary Material accompanying Cha (2023a). Reproduced from Cha (2023a), licensed under the Creative Commons Attribution 4.0 International License (CC BY 4.0).

Across environments, features were held constant except for the target manipulation, with one planned exception: the window-view classrooms (city and nature) included two small windows on the left wall, whereas the classroom-size environments (large and small) did not include these side windows. In the classroom-size set, the small room could not accommodate the same amount of furniture and play materials as the large room; however, to minimize confounding, the core activity areas and essential materials were kept equivalent across size conditions, and only non-essential peripheral furnishings were omitted in the small-room version. Accordingly, the classroom-size comparison should be understood as a realistic size manipulation with necessarily adapted furnishings rather than as a perfectly isolated geometric scaling of an otherwise identical room. Floor plans are provided in Figure 2.

#### 2.2.3 Experimental procedures

Parents (primarily mothers) registered through an online Google Forms survey. Applicants were first stratified by the child’s sex (boy vs. girl) and then randomly assigned to one of the four VR classroom environments. Parents received an overview of the study via telephone including the study purpose, procedures, compensation, the voluntary nature of participation, and the child’s right to discontinue participation at any time, and laboratory appointments were scheduled. A reminder message containing the visit information was sent 1 day prior to the appointment.

As noted above, the dataset analyzed in this manuscript was collected as part of a broader study that included additional measures not examined here (see Cha, 2023a). Accordingly, upon arrival at the laboratory, caregivers again received an explanation of the study purpose, procedures, compensation, and the child’s right to discontinue participation at any time without penalty, after which written informed consent was obtained prior to data collection. Children were addressed in age-appropriate language and told that they could stop the VR experience whenever they felt dizzy, uncomfortable, or simply did not wish to continue. Caregivers were allowed to remain nearby and observe the procedure. During the same visit, children also completed the broader study protocol, which included saliva sampling for cortisol assessment before and after the VR session and computer-based tasks assessing executive functioning; these measures are not analyzed in the present study. Caregivers completed questionnaires on child background characteristics (e.g., age in months, sex, and preschool enrollment/experience). To protect privacy, identifying information was stored separately from the analytic dataset and all analyzed records were linked only by participant ID code.

Each child first sat on a chair positioned at the center of the physical 2.3 m × 2.3 m square area and then wore the headset. After headset fitting, each child completed the built-in five-point eye-tracking calibration provided by the VIVE Pro Eye/SRanipal system prior to VR exposure. Following the standard device routine, participants were instructed to visually follow the on-screen calibration targets while the headset position and IPD were adjusted as needed. Calibration was completed before exposure to the classroom scene began. Children were then briefly exposed to a neutral virtual corridor for approximately 1 min to ensure comfort in the virtual environment and to confirm adequate adaptation to the virtual space and basic controller use. After this adaptation period, and once no apparent difficulties were observed, the experimenter initiated the classroom scene and positioned each child at a standardized seated starting point located at the center of the classroom. All children began the classroom session from the same central starting location and with the same initial facing direction—toward the front wall containing the central window, opposite the door (Figure 2).

The VR classroom experience lasted 10 min and consisted of two phases: Phase 1 involved 5 min of seated exploration from this standardized central starting position, and Phase 2 involved 5 min of free movement. In Phase 1, children remained seated on a backless chair and were invited to look around the classroom freely while seated; no additional task goal was given. In Phase 2, children were allowed to move around the virtual classroom freely by walking and/or using the controller. This starting configuration was selected because the broader project focused on children’s exposure to classroom physical conditions (e.g., window-view content and classroom size), rather than on doorway-entry behavior per se. During pilot testing, we observed that when children were seated on a chair with a backrest, their ability to rotate freely and inspect the classroom in all directions was constrained. We therefore adopted a backless chair together with a common central starting point so that children could turn more easily and have broad and comparable 360° visual access to the classroom while seated. The present manuscript analyzes Phase 1 only (0–300 s), because this interval captures early visual orientation under a standardized seated starting condition before locomotion begins. The study protocol was approved by the University Bioethics Review Committee (Approval No. 1044396-202007-HR-138-04).

### 2.3 Measures

#### 2.3.1 Areas of interest (AOI)

To determine which Area of Interest (AOI) the child was viewing at each time point, we used a raycasting approach within Unity. A virtual ray was projected along the gaze-direction vector, and the intersected object or functional area was recorded as the AOI for that frame. AOI boundaries were specified a priori in the 3D scene and AOI assignment was generated algorithmically from gaze-ray intersections with predefined Unity colliders. This procedure enabled the mapping of raw gaze vectors to interpretable functional regions (e.g., Block area, Book area) for subsequent transition-based analyses.

Gaze mapping was first performed at the object level (e.g., chair, table, shelf) and then aggregated into a higher-level AOI taxonomy reflecting functional classroom activity areas. The final AOI scheme consisted of seven core areas: Block, Science, Manipulatives, Book, Dramatic Play, Art, and Music (Figure 2). We adopted a functional-zone taxonomy because all children were enrolled in early childhood education settings (enrollment duration in months: Mean = 51.89, SD = 13.32), and therefore were familiar with classroom area structures and likely to perceive sets of objects and materials as unified functional regions. Aggregating gaze from individual objects to functional areas allowed the analytic unit to correspond more closely to how the environment was arranged and how children were likely to encounter and interpret it in everyday classroom experience.

In addition to the seven activity areas, we coded key structural elements relevant to early orientation. “Window” was subdivided into a central (front-facing) window and small side windows. The frontal wall area surrounding the central window was coded as the Fringe Wall, allowing us to distinguish gaze directed at the window itself from gaze directed at the adjacent frontal wall region. Wall fixations within an activity area were assigned to the corresponding activity zone. Other non-activity structural elements (e.g., ceiling, lights, doors, corridor surfaces) were coded as “Environment.” Gaze samples that were not meaningfully attributable to functional regions (e.g., transient intersections with gaps, floor fragments, or tracking-related artifacts) were coded as “Empty.” Because Empty was treated as non-informative and not central to the research questions, it was excluded from primary results reporting.

Importantly, window configuration differed across the two experimental contrasts. In the classroom-size environments, no side windows were present beyond the frontal window. In the window-view environments, however, the window complex consisted of a large front-facing central window plus two small windows on the left wall (Figures 1, 2). Therefore, for window-view analyses we separately quantified gaze allocation to (a) the total window complex and (b) its components (central vs. small windows), to examine whether children primarily accessed the outdoor scene through the central opening or through the smaller side windows. This distinction was necessary because outdoor view content was the focal manipulated feature in the window-view comparison, and the interpretation depends on how children distributed window-directed gaze across window elements.

### 2.4 Analysis

This study employed a between-subjects design, in which each child was randomly assigned to one of four VR classroom conditions (city-view, nature-view, large classroom, small classroom). Analyses were conducted separately for two contrasts: (a) the window-view comparison and (b) the classroom-size comparison. All analyses focused exclusively on gaze data from the initial 5 min (0–300 s, Phase 1) of standardized seated exposure to the classroom scene. Prior VR studies with children have typically used exposure durations of approximately 2.5–3 min (Sathyanarayanan et al., 2024); however, given the relatively young age of participants, the observation window was extended to 5 min to more fully capture early orientation and adaptation processes. Moreover, because the VR protocol consisted of 5 min seated followed by 5 min of free movement, restricting analyses to the seated phase (Phase 1) ensured procedural and behavioral consistency.

### 2.4.1 AOI-based proportional gaze analysis

For each participant, total dwell time within each AOI was computed and converted into a proportion of total valid gaze time (%). These proportions were used to characterize overall attentional allocation patterns across conditions. Normality (Shapiro–Wilk) and homogeneity of variance (Levene’s test) were assessed prior to inferential comparisons. When assumptions were met, independent-samples *t*-tests were conducted (using Welch’s correction when variances were unequal). When assumptions were violated, Mann–Whitney U-tests were used.

### 2.4.2 Transition-based preprocessing

To examine exploration from a process-oriented perspective, we derived transition-based metrics from AOI sequences. First, gaze samples were converted into temporally ordered AOI sequences. Consecutive repetitions of the same AOI were collapsed via run-length encoding, such that each uninterrupted visit to an AOI was treated as a single state. This ensured that analyses captured transitions between regions rather than sample-level dwell time. Second, samples coded as Empty were removed for transition computation. A bridging rule was applied: if an Empty segment occurred between two AOIs, the transition was computed directly between the preceding and following valid AOIs (e.g., Block → Empty → Window_Central was treated as Block → Window_Central). For each participant, transition counts N*_*i*_*_→_*_*j*_* were computed, and transition probabilities were defined as:


$$p\left(j|i\right)=\frac{N_{i\to j}}{\sum_{k}N_{i\to k}}$$

where the denominator represents the total number of outgoing transitions from AOI *i*.

### 2.4.3 Window-view condition: transition metrics

Because the window-view manipulation altered outdoor content while preserving interior layout, we focused on whether children’s exploration was structured around the window as an anchor or whether gaze expanded outward into interior regions. Windows were divided into two components: Window_Central and Window_Small. Six participant-level metrics were computed and standardized (z-scores) prior to clustering:

#### 2.4.3.1 Outgoing entropy from windows

For each window *w*, outgoing transition entropy was computed as:


$$H\left(w\right)=-\sum_{a}p\left(a|w\right)\log p\left(a|w\right)$$

Entropy was normalized to allow comparability. Higher entropy indicates a more diverse distribution of destinations. To ensure comparability across participants, the destination set was fixed to the top-K most frequent targets (*K* = 5) identified from pooled data.

#### 2.4.3.2 Average return rate

To capture the tendency to return to a window after viewing other regions, we computed the probability that gaze from major non-window AOIs transitioned to either window type. Major non-window AOIs were defined as the five highest-outflow non-window regions in pooled data. Return rates were aggregated as weighted means based on outflow frequency.

#### 2.4.3.3 Branch central ratio

Given a return to any window, we calculated the conditional probability that the return was directed to the central window:

Branch Central Ratio = P(Central Window | Return to any window)

#### 2.4.3.4 Average anchor return (l ≤ 3 excursions)

An excursion was defined as leaving window *w* and subsequently returning to the same window. Short excursions were limited to ≤ 3 intermediate AOIs(e.g., *w*→A→*w* = 1, *w*→A→B→*w* = 2, *w*→A→B→C→*w* = 3). The constraint (L ≤ 3) ensured stability within the 5-min observation window and aligned with a theoretically interpretable definition of anchor behavior.

#### 2.4.3.5 Average expansion (two-step expansion)

To capture outward expansion from windows, we defined 2-step sequences, *w→h→x*, where *h* is one of the top-3 most frequent hubs following window departure, and *x* is a non-window AOI. Expansion rates were weighted by hub frequency and averaged across window types.

#### 2.4.4 Classroom-size condition: dynamic exploration metrics

##### 2.4.4.1 Switch rate

If the compressed AOI sequence has length *T*, then:


Switch⁢Count=T- 1

$$\mathrm{Switch}\,\mathrm{Rate}=\frac{T-1}{t_{v\,a\,l\,i\,d}}$$

##### 2.4.4.2 Median state duration

The median dwell duration across AOI visits was used to summarize temporal persistence.

##### 2.4.4.3 Transition entropy

State-level entropy *H*(*i*) was computed as above, and overall transition entropy was calculated as a weighted average:


$$H=\sum_{i}p\,\left(i\right)\,H\,\left(i\right)$$

Higher values indicate a more diverse and complex transition structure.

#### 2.4.5 Clustering and prediction of individual characteristics

Based on the derived metrics, we conducted *k*-means clustering separately for each contrast: (a) the window-view comparison pooled City and Nature participants, and (b) the classroom-size comparison pooled Large and Small participants. The number of clusters (*k*) was determined using a combination of the silhouette coefficient, the Calinski–Harabasz (CH) index, the Davies–Bouldin (DB) index, and the minimum cluster size to avoid unstable small clusters. To explore whether cluster membership was associated with individual characteristics, we fitted binary logistic regression models when the solution yielded two clusters, and multinomial logistic regression models when three or more clusters were retained (or, when warranted, exploratory comparisons focusing on major clusters given sample size and cluster-size stability). Predictors included child sex (boy = 0, girl = 1), age in months, and enrollment duration in months, and models were estimated via maximum likelihood.

Finally, we verified demographic equivalence across conditions. The city- and nature-view groups did not differ in sex distribution, χ^2^(1) = 0.00, *p* = 1.00, age in months, *t* = −1.15, *p* = 0.25, or enrollment duration, *t* = 0.20, *p* = 0.84. Likewise, large- and small-classroom groups did not differ in sex distribution, χ^2^(1) = 0.16, *p* = 0.98, age in months, *t* = −0.75, *p* = 0.45, or enrollment duration, *t* = 0.04, *p* = 0.97.

All data processing and analyses—including gaze preprocessing, transition matrix construction, metric computation, clustering, and inferential statistics—were conducted in Python.

## 3 Results

### 3.1 Window view condition: city- vs. nature-view

#### 3.1.1 Overall gaze-allocation patterns

**FIGURE 3:**
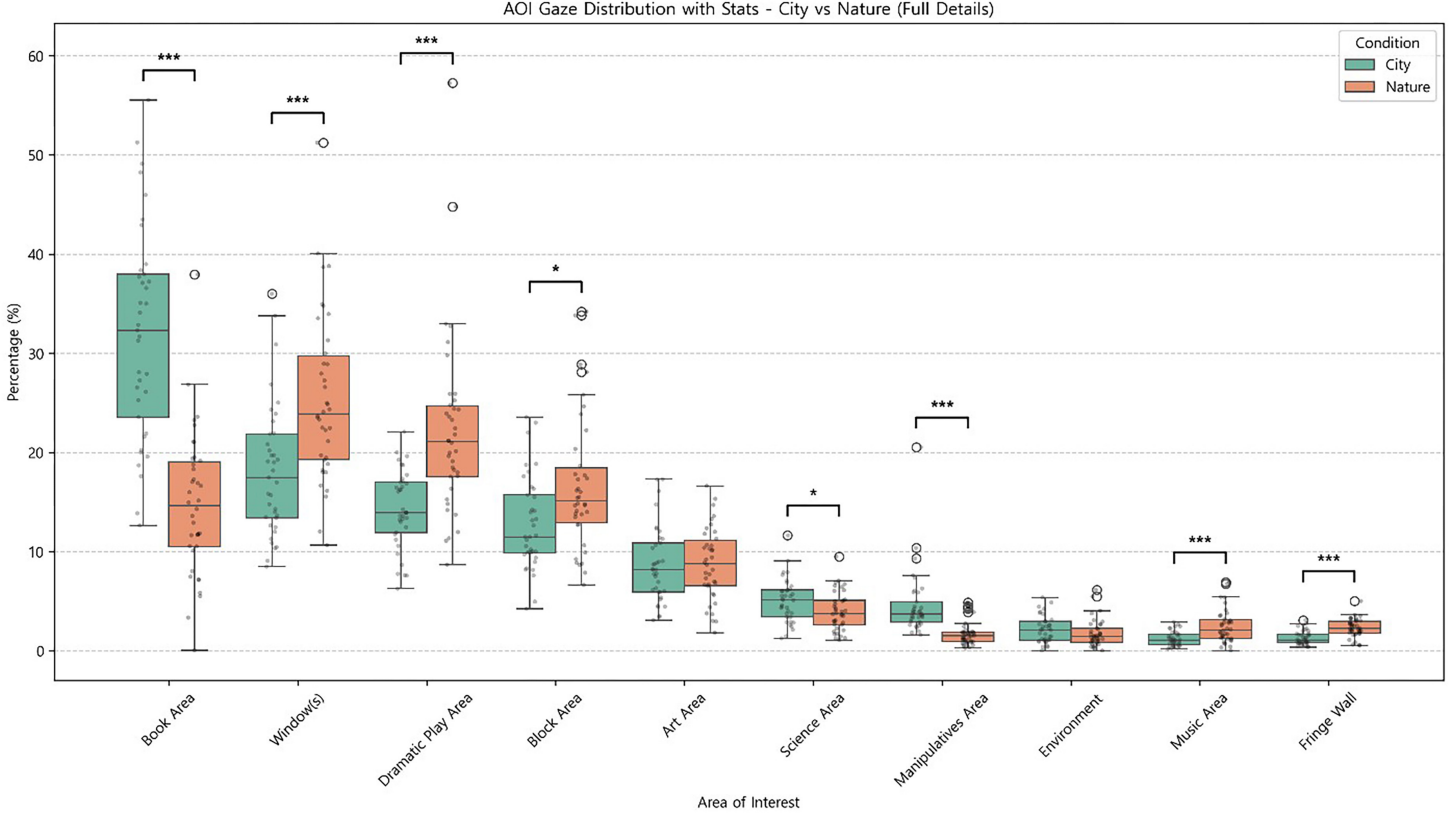
Proportional gaze time by AOI (0–300 s): City-view vs. Nature-view. *p < 0.05, ***p < 0.001.

Comparisons of AOI-level proportional gaze time (Figure 3) indicated robust differences in both the relative magnitude and distribution of attention across regions between the nature-view and city-view conditions. Specifically, children in the city-view condition allocated a significantly greater proportion of gaze to the Book Area than did children in the nature-view condition (City: *M* = 31.99%, Nature: *M* = 14.75%), Welch’s *t* = 8.13, *p* < 0.001. In contrast, children in the nature-view condition showed significantly greater gaze allocation to the window complex (central and small windows combined; City: *M* = 18.12%, Nature: *M* = 25.43%), Mann–Whitney *U* = 339, *p* < 0.001; the Dramatic Play Area (City: *M* = 14.25%, Nature: *M* = 22.20%), *U* = 245, *p* < 0.001; and the Block Area (City: *M* = 12.77%, Nature: *M* = 16.68%), *U* = 465, *p* = 0.012, compared with the city-view condition. Among the remaining activity zones, the Science Area received significantly greater attention in the city-view condition (City: *M* = 5.05%, Nature: *M* = 3.97%), *t* = 2.26, *p* = 0.027, as did the Manipulatives Area (City: *M* = 4.62%, Nature: *M* = 1.72%), *U* = 1,251, *p* < 0.001. Conversely, the Music Area (City: *M* = 1.19%, Nature: *M* = 2.47%), *U* = 349, *p* < 0.001, and the Fringe Wall Area (City: *M* = 1.26%, Nature: *M* = 2.31%), *U* = 255, *p* < 0.001, received significantly greater attention in the nature-view condition, although these AOIs accounted for only a small proportion of overall gaze time. Gaze to the Art Area did not differ between conditions (City: *M* = 8.64%, Nature: *M* = 8.74%), *t* = −0.12, *p* = 0.901, and gaze to the Environment also did not differ significantly (City: *M* = 2.11%, Nature: *M* = 1.74%), *U* = 824.5, *p* = 0.200 (Figure 3).

**FIGURE 4:**
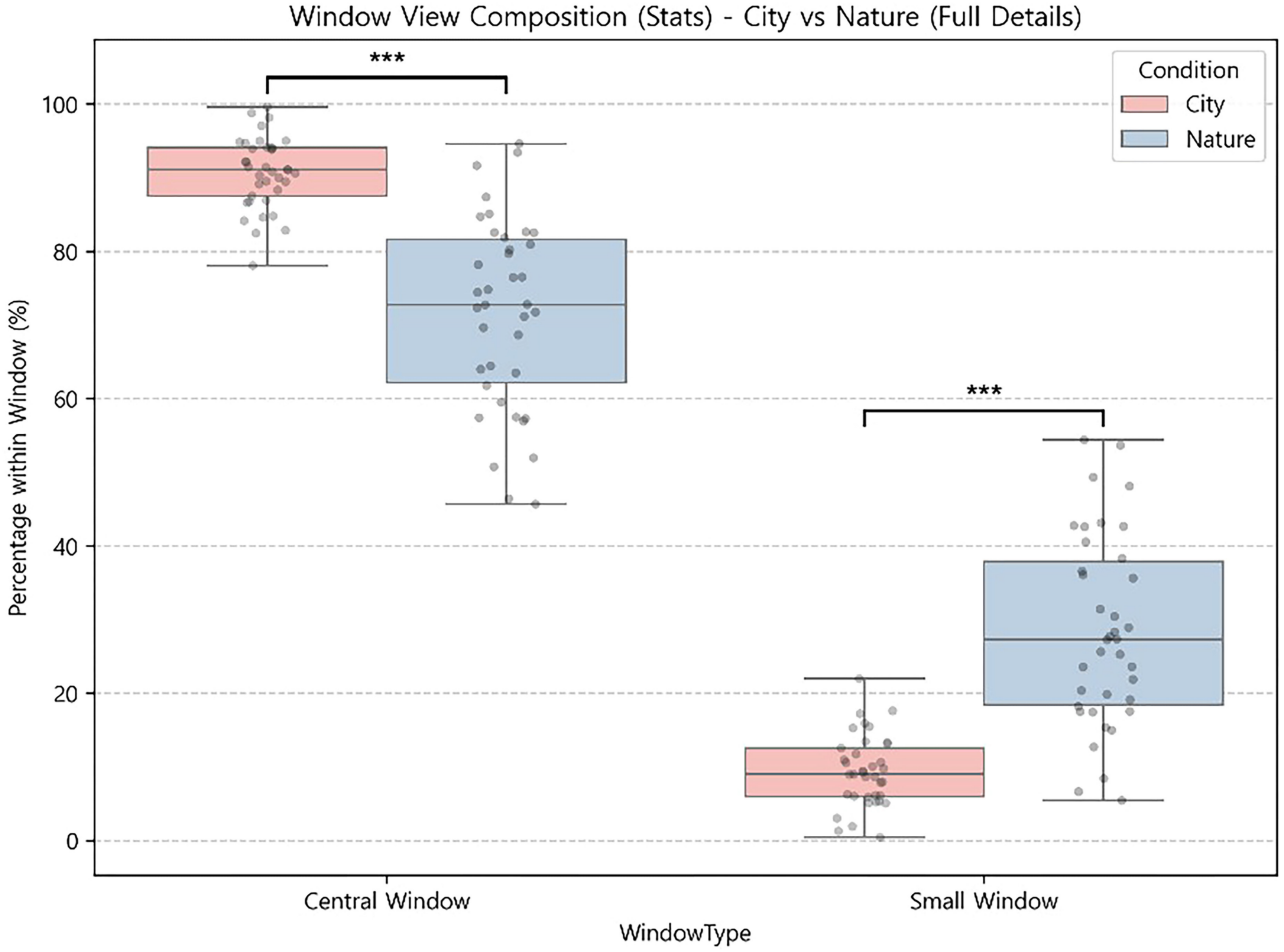
Within-window gaze allocation (central vs. side windows) conditional on window-directed gaze: City-view vs. Nature-view. ***p <0.001.

To further characterize window-directed attention, we decomposed gaze within the window complex and compared the proportion directed to the central window versus small window(s) (Figure 4). The two conditions differed significantly in the composition of window-directed gaze. In the city-view condition, a larger share of window-directed gaze was concentrated on the central window (City: *M* = 90.66%, Nature: *M* = 71.65%), Welch’s t = 8.47, *p* < 0.001, whereas the share directed to small windows was correspondingly lower (City: *M* = 9.34%, Nature: *M* = 28.35%), Welch’s *t* = −8.47, *p* < 0.001. Thus, window-directed attention in the city-view condition was more strongly centralized, whereas the nature-view condition showed a more distributed pattern of window sampling across openings (Figure 4).

Notably, these within-window differences align with the broader AOI allocation patterns. In the nature-view condition, higher gaze allocation to the small windows coincided with greater attention to nearby regions, particularly the Block Area and Dramatic Play Area, which are spatially adjacent to the side windows. In contrast, in the city-view condition, the strong concentration on the central window co-occurred with relatively greater attention to the Science, Manipulatives, and Book areas located to the right of the central window from the child’s viewpoint. Taken together, these results suggest that the window complex and its associated outdoor view functioned as a salient organizing feature in preschoolers’ early visual exploration, shaping both which classroom regions attracted attention and how window-directed gaze was distributed across window elements.

Overall, gaze allocation during the first 5 min showed condition-specific exploration profiles. The city-view condition was characterized by relatively greater attention to the Book Area and a pronounced central-window–focused pattern. The nature-view condition was characterized by greater attention to the window complex overall, a higher relative contribution of small-window viewing, and comparatively greater attention to adjacent activity areas (particularly Dramatic Play and Blocks).

#### 3.1.2 RQ1: how does early classroom exploration differ by window view condition (city vs. nature) in transition-based clusters?

To characterize preschoolers’ early gaze-transition organization under the window-view manipulation (City vs. Nature), we conducted a *k*-means cluster analysis using six process-oriented transition metrics computed from AOI sequences during the first 300 s. These metrics captured: (a) outgoing transition entropy from the central window, (b) outgoing transition entropy from the small side windows, (c) return-to-window rate from major non-window areas, (d) the conditional branching ratio toward the central window given a window return, (e) anchor-return probability to the same window within short excursions, and (f) a two-step window-based expansion rate. All metrics were *z*-standardized prior to clustering.

Candidate solutions from *k* = 2 to *k* = 6 were evaluated using the silhouette coefficient, Calinski–Harabasz (CH) index, Davies–Bouldin (DB) index, and minimum cluster size (Table 1). The two-cluster solution provided the best overall balance of separation and stability, yielding the highest silhouette (0.326) and CH (40.03) values while maintaining adequate cluster sizes (*n* = 44 and *n* = 30). In contrast, solutions with *k* > 2 showed reduced separation and produced small clusters (minimum *n* ≤ 8), suggesting over-partitioning without commensurate gains in interpretability. Accordingly, we retained the two-cluster solution as the most parsimonious representation of window-based transition strategies.

**TABLE 1:** Model fit indices for *k*-means clustering of window-view transition metrics (*k* = 2–6)

Number of clusters (k)	Silhouette coefficients	Calinski–Harabasz index	Davies–Bouldin index	Min N	Max N
2	0.326	40.03	1.178	30	44
3	0.297	31.5	1.169	8	39
4	0.282	31.04	1.216	6	27
5	0.251	27.87	1.209	6	25
6	0.23	25.86	1.268	6	18

Min N indicates the smallest cluster size, while Max N indicates the largest cluster size.

**FIGURE 5:**
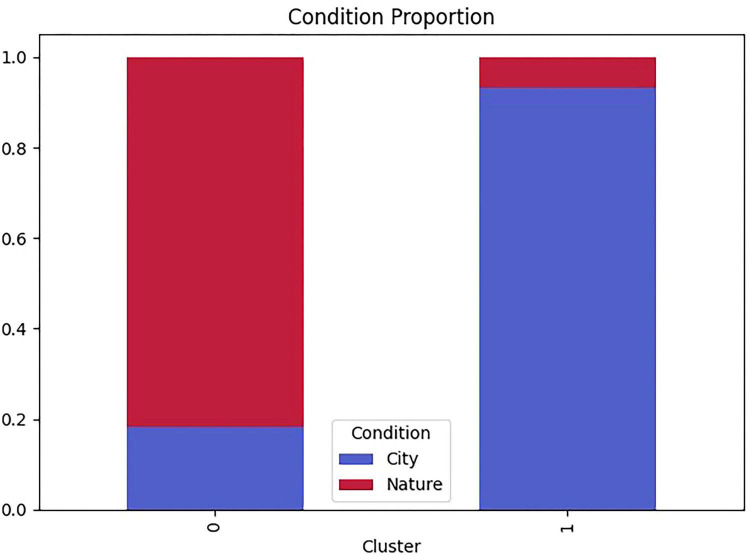
Distribution of transition-based exploration clusters by window-view condition.

Cluster membership differed substantially by window-view condition (Figure 5 and Table 2). A chi-square test indicated a strong association between condition and cluster assignment, χ^2^(1) = 37.37, *p* < 0.001, Cramér’s *V* = 0.71. Most children in the nature-view condition were classified into Cluster 0 (94.7%), whereas most children in the city-view condition were classified into Cluster 1 (77.8%).

**TABLE 2:** Window-view exploration clusters: descriptive statistics and cluster centroids for transition metrics.

Cluster	Interpretive label	N	Central out entropy	Small out entropy	Average return rate	Branch central ratio	Average anchor return	Average expansion
0	Return-anchored/stabilization	44 (City = 8, Nature = 36)	0.692 (0.08)	0.542 (0.11)	0.431 (0.09)	0.609 (0.12)	0.653 (0.07)	0.341 (0.05)
1	Expansion-oriented/exploratory	30 (City = 28, Nature = 2)	0.730 (0.06)	0.563 (0.17)	0.281 (0.05)	0.731 (0.08)	0.489 (0.08)	0.544 (0.08)
*F*(1, 72)	–	–	5.08	0.46	78.33	25.99	92.05	189.21
*p*	–	–	0.027	0.500	< 0.001	<0.001	< 0.001	<0.001
η^2^	–	–	0.066	0.006	0.521	0.265	0.561	0.724

Values are Mean (SD) of participant-level transition metrics computed from AOI sequences during the first 300 s. Central Out Entropy: higher values indicate more diverse destinations when leaving the central window; Small Out Entropy: higher values indicate more diverse destinations when leaving the small side windows; Average Return Rate: higher values indicate a stronger tendency to return to any window (central or small) on the next transition after looking at major non-window areas (computed as an outflow-weighted mean across those areas); Branch Central Ratio: higher values indicate a stronger central-window bias conditional on a window return (i.e., given a return to any window, the probability that the return lands on the central window rather than the small windows); Average Anchor Return: higher values indicate stronger anchoring to the same window, where an anchor return is counted when gaze leaves a window and returns to the same window within three or fewer intermediate AOI transitions; Average Expansion: higher values indicate stronger outward exploration beyond the windows, operationalized as a higher proportion of two-step sequences (*w→h→x*) that end in a non-window AOI (excluding Empty).

**FIGURE 6:**
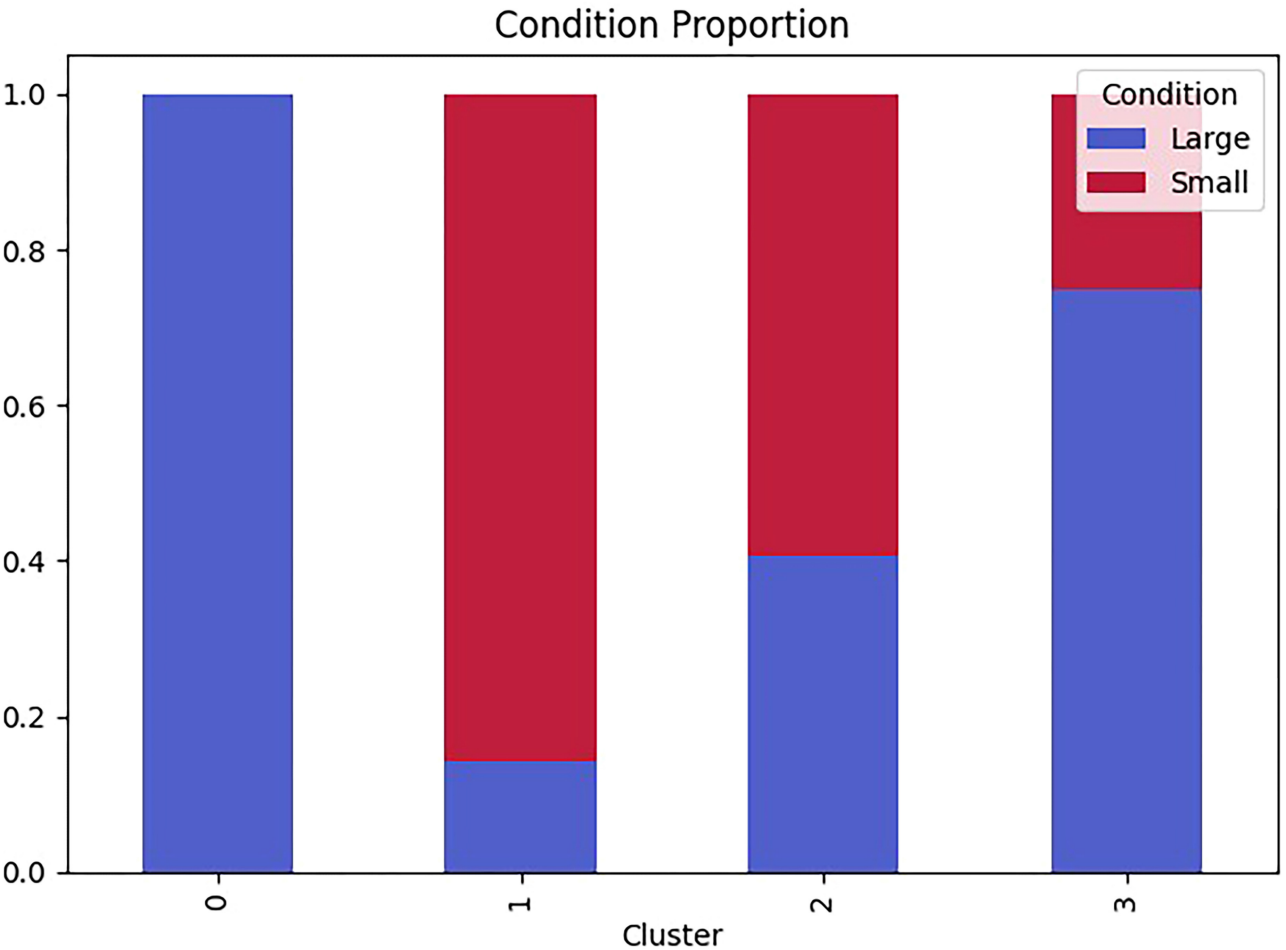
Standardized transition metrics (z-scores) by window-view exploration cluster.

To verify that the two clusters were meaningfully differentiated on the six clustering features, we conducted one-way ANOVAs (df = 1, 72) for each transition metric using cluster as the grouping factor. Results indicated significant between-cluster differences on five of the six metrics, with the exception of Small Out Entropy, which did not differ between clusters, *F*(1, 72) = 0.46, *p* = 0.500, η^2^ = 0.006. Significant differences were observed for Central Out Entropy, *F*(1, 72) = 5.08, *p* = 0.027, η^2^ = 0.066; Average Return Rate, *F*(1, 72) = 78.33, *p* < 0.001, η^2^ = 0.521; Average Anchor Return, *F*(1, 72) = 92.05, *p* < 0.001, η^2^ = 0.561; Branch Central Ratio, *F*(1,72) = 25.99, *p* < 0.001, η^2^ = 0.265; and Average Expansion, *F*(1, 72) = 189.21, *p* < 0.001, η^2^ = 0.724. Standardized (z-score) comparisons further illustrated these profile differences across metrics (Figure 6).

The two clusters reflected qualitatively distinct transition-organization patterns (Table 2). Cluster 0 was characterized by stronger window re-orientation and anchoring (higher return-to-window rate and higher anchor-return probability) combined with lower two-step expansion, consistent with a return-anchored/stabilization pattern. Cluster 1, by contrast, showed reduced window returns and anchoring but substantially higher two-step expansion and a stronger conditional bias toward returning to the central window, consistent with an expansion-oriented/exploratory pattern.

Taken together, these results suggest that the outdoor scene visible through the windows reorganized not only where children looked but also the temporal structure of their gaze transitions—favoring a window-return–anchored pattern in the nature-view condition and a more expansion-oriented pattern in the city-view condition.

#### 3.1.3 RQ2: do child characteristics predict transition-based cluster membership within each window-view condition?

To examine whether children’s individual characteristics (sex, age in months, and preschool enrollment duration) predicted membership in the transition-based exploration clusters, we fitted separate maximum-likelihood logistic regression models for the city-view group (*n* = 36) and the nature-view group (*n* = 38) (Tables 3, 4). In each model, the dependent variable was cluster membership (Cluster 0 vs. Cluster 1) derived from the pooled window-view clustering solution, and predictors included sex (boy = 0, girl = 1), age (months), and enrollment duration (months).

**TABLE 3:** Logistic regression predicting window-view cluster membership (City-view condition).

Predictor	B (SE)	OR	95% CI for OR	*p*
Sex	0.050 (0.874)	1.05	[0.19, 5.84]	0.954
Age in months	−0.057 (0.098)	0.95	[0.78, 1.15]	0.564
Enrollment in months	0.024 (0.034)	1.02	[0.96, 1.09]	0.474

**TABLE 4:** Logistic regression predicting window-view cluster membership (Nature-view condition).

Predictor	B (SE)	OR	95% CI for OR	*p*
Sex	−0.102 (1.485)	0.90	[0.05, 16.56]	0.945
Age in months	−0.002 (0.129)	1.00	[0.78, 1.28]	0.989
Enrollment in months	−0.008 (0.050)	0.99	[0.90, 1.09]	0.867

In the city-view condition, the overall model fit did not differ significantly from the intercept-only model (LLR *p* = 0.875; Pseudo *R*^2^ = 0.018), and none of the predictors significantly predicted cluster membership (sex: *p* = 0.954; age: *p* = 0.564; enrollment duration: *p* = 0.474). Likewise, in the nature-view condition, the model also failed to demonstrate a significant improvement over the intercept-only model (LLR *p* = 0.998; Pseudo *R*^2^ = 0.002), and no predictors were significantly associated with cluster membership (sex: *p* = 0.945; age: *p* = 0.989; enrollment duration: *p* = 0.867). Because cluster membership was highly imbalanced within each view condition, coefficient estimates may be unstable (quasi-separation); therefore, these models are interpreted as exploratory and primarily descriptive of trends rather than confirmatory tests.

### 3.2 Classroom size condition: large vs. small classroom

#### 3.2.1 Overall gaze-allocation patterns

**FIGURE 7:**
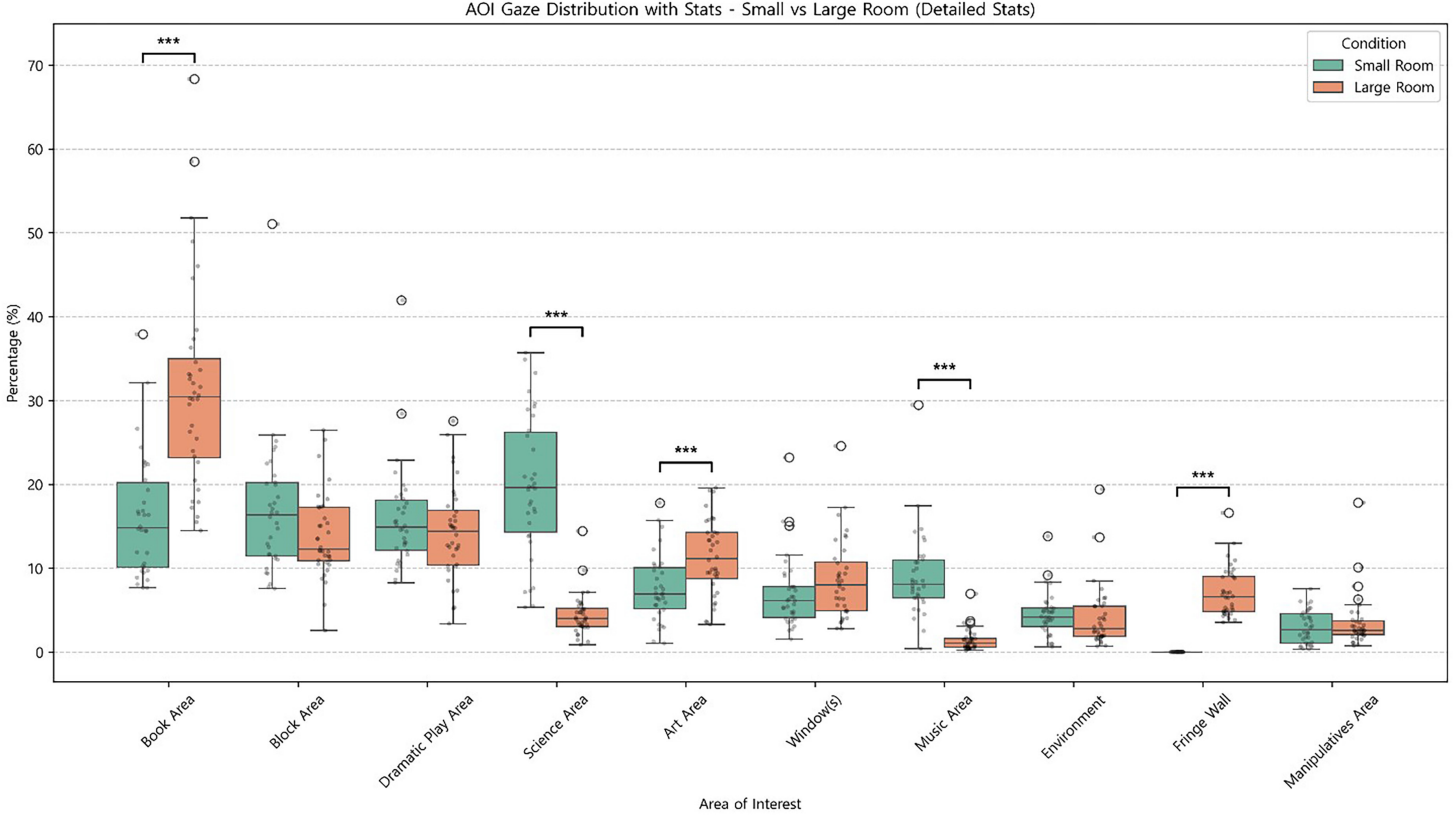
Proportional gaze time by AOI (0-300s): Small vs. Large classroom. ***p <0.001.

AOI-level proportional gaze time differed across the classroom-size conditions (Figure 7), with several regions showing systematic shifts in attentional allocation. In the large classroom, the Book Area received the highest proportion of gaze (*M* = 31.39%), which was significantly greater than in the small classroom (*M* = 16.14%), Mann–Whitney *U* = 144, *p* < 0.001. In contrast, in the small classroom, the Science Area received the greatest proportion of gaze (*M* = 19.84%), exceeding the allocation observed in the large classroom (*M* = 4.45%), *U* = 1,196, *p* < 0.001. The Music Area likewise drew greater attention in the small classroom (Small: *M* = 9.11%; Large: *M* = 1.41%), *U* = 1176, *p* < 0.001, whereas the Art Area received a greater proportion of gaze in the large classroom (Large: *M* = 11.31%; Small: *M* = 7.59%), *t*(67) = −3.63, *p* < 0.001. Notably, the Fringe Wall (i.e., the front-wall region surrounding the central window) showed an especially large size-related difference: gaze to the Fringe Wall was nearly absent in the small classroom (*M* = 0.00%) but was clearly present in the large classroom (*M* = 7.14%), *U* = 0, *p* < 0.001. In contrast, proportional gaze to Window(s) (Small: *M* = 7.03%; Large: *M* = 8.66%), *U* = 465, *p* = 0.085; the Block Area (Small: *M* = 16.93%; Large: *M* = 13.81%), *U* = 754, *p* = 0.096; the Dramatic Play Area (Small: *M* = 15.90%; Large: *M* = 14.23%), *U* = 696, *p* = 0.326; the Manipulatives Area (Small: *M* = 2.98%; Large: *M* = 3.51%), *U* = 568, *p* = 0.609; and the Environment (Small: *M* = 4.48%; Large: *M* = 4.11%), *U* = 724, *p* = 0.190 differed descriptively but did not reach statistical significance.

Inspection of the overall AOI distribution suggests that the reduced salience of the frontal window in the classroom-size environments—where the window occupied roughly half of the front wall rather than most of it—was accompanied by a lower overall level of window-directed gaze relative to the window-view environments. Within this context, the large classroom was characterized by a strong emphasis on the Book Area, followed by relatively greater attention to zones located primarily along the left wall. By contrast, the small classroom showed a more distributed exploration pattern centered on the Science Area, with relatively greater attention allocated across multiple regions (e.g., Block, Book, and Dramatic Play). Unlike the other three environments (city-view, nature-view, and large classroom), the small classroom also showed comparatively higher gaze allocation to the Music Area. A supplementary audit of the standardized initial field of view further showed that the Science Area occupied a larger proportion of the initial scene in the small classroom (9.38%) than in the large classroom (4.68%), suggesting that greater initial geometric prominence might have contributed to the elevated Science-area gaze in the small-room condition.

Taken together, these findings suggest that when a highly dominant frontal attractor such as a wall-sized central window is absent, the strong front-facing orientation pattern observed in the window-view environments is attenuated. Moreover, classroom size was associated with different early prioritization profiles: the large classroom showed relatively greater attention to the Book Area (and, descriptively, to several activity zones including Dramatic Play and Blocks), whereas the small classroom showed relatively greater attention to Science and Music and a more distributed allocation across several activity zones. These systematic differences in early gaze allocation suggest that spatial scale may shape which areas become primary targets of attention during initial classroom orientation. Accordingly, whereas the window-view comparison motivated attention to anchoring and outward expansion around the window, the classroom-size comparison motivates a complementary focus on process-level exploration dynamics—including how frequently children switch between areas (switch rate), how long they dwell within a region (duration), and how concentrated versus diverse their transition structure is (transition entropy).

#### 3.2.2 RQ3: how does early classroom exploration differ by classroom size (large vs. small) in process-level exploration clusters?

To classify preschoolers’ early classroom exploration profiles in the classroom-size comparison (Large vs. Small), we conducted *k*-means clustering based on three process-oriented metrics derived from AOI state sequences during the first 5 min (0–300 s): Switch Rate, Median State Duration, and Transition Entropy. Candidate solutions (*k* = 2–8) were evaluated using the silhouette coefficient, the Calinski–Harabasz (CH) index, the Davies–Bouldin (DB) index, and minimum cluster size (Table 5). The four-cluster solution was selected based on the strongest overall balance among separation, compactness, and minimum cluster size, yielding the highest silhouette (0.401) and CH (52.273) values and the lowest DB value (0.812). For *k* ≥ 5, indices deteriorated overall and the minimum cluster size fell to ≤ 6, raising concerns about stability and interpretability. Accordingly, the four-cluster solution was adopted. To assess the stability of the selected four-cluster solution, supplementary bootstrap analyses were conducted. When candidate solutions from *k* = 2–11 were considered, *k* = 4 was selected most frequently (81.6% of 1,000 bootstrap resamples). In addition, when bootstrap resampling was performed with *k* fixed at 4, the defining profile of each cluster re-emerged, including Cluster 3, whose recovered size varied (*M* = 9.31, SD = 3.89; 95% bootstrap interval = 4–20) but preserved its characteristic combination of very high switching and very short state duration. Taken together, these analyses supported retention of the *k* = 4 solution.

**TABLE 5:** Model fit indices for k-means clustering of classroom-size exploration metrics (*k* = 2–8).

Number of clusters (k)	Silhouette coefficient	Calinski–Harabasz index	Davies–Bouldin index	Inertia	Min N
2	0.326	36.993	1.058	133.365	19
3	0.354	43.603	1.034	89.174	14
4	0.401	52.273	0.812	60.658	8
5	0.317	48.692	0.994	51.196	6
6	0.287	43.204	1.044	46.738	7
7	0.298	42.588	1.038	40.419	6
8	0.296	39.929	0.986	37.084	4

Indices were computed from standardized process-level exploration metrics (Switch Rate, Median State Duration, Transition Entropy) over 0–300 s. *minN* indicates the smallest cluster size.

**FIGURE 8:**
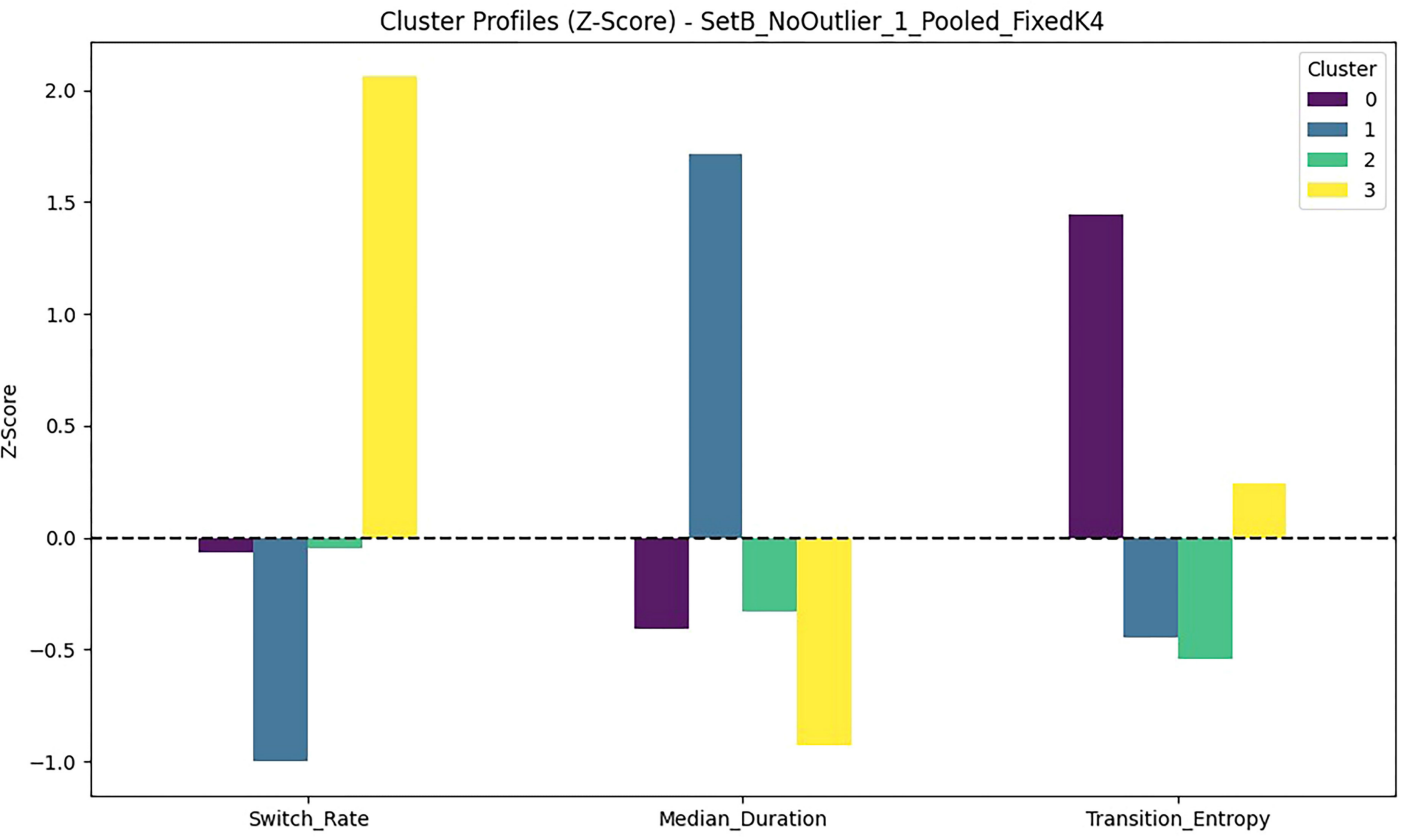
Distribution of process-level exploration clusters by classroom-size condition.

**FIGURE 9:**
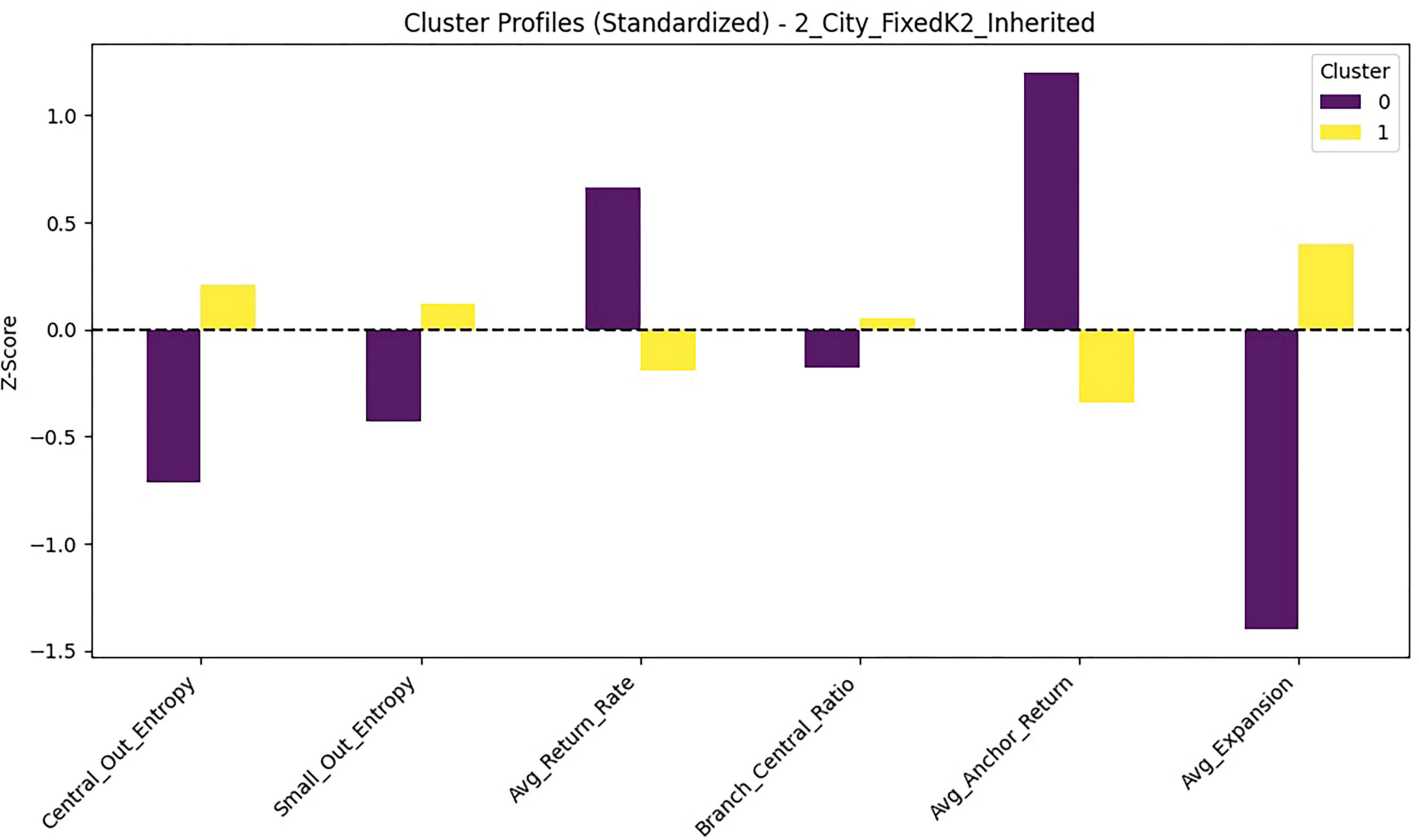
Standardized process-level exploration metrics (z-scores) by classroom-size exploration cluster.

To confirm that the four clusters were meaningfully differentiated on the clustering features, we conducted one-way ANOVAs (df = 3, 65) with cluster as the grouping factor and each metric as the dependent variable. All three metrics differed significantly across clusters (Figure 8 and Table 6), indicating that the identified profiles represented statistically separable exploration rhythms and transition structures. Specifically, clusters differed on Switch Rate, *F*(3, 65) = 51.35, *p* < 0.001, η^2^ = 0.703; Median State Duration, *F*(3, 65) = 73.61, *p* < 0.001, η^2^ = 0.773; and Transition Entropy, *F*(3, 65) = 38.34, *p* < 0.001, η^2^ = 0.639. Standardized (z-score) comparisons further illustrated these profile differences (Figure 9).

**TABLE 6:** Classroom-size exploration clusters: descriptive statistics and cluster centroids for dynamic metrics.

Cluster	Interpretive label	N	Switch rate	Median State duration (s)	Transition entropy
0	Moderate–diverse (mid switch, high entropy)	15(Large = 15, Small = 0)	1.722 (0.329)	0.117 (0.039)	1.241 (0.061)
1	Moderate–Repetitive (mid switch, low entropy)	33 (Large = 13, Small = 20)	1.728 (0.313)	0.127 (0.040)	0.994 (0.067)
2	Slow–Focal (low switch, long dwell, low entropy)	13 (Large = 2, Small = 11)	1.176 (0.254)	0.294 (0.046)	1.000 (0.090)
3	Rapid–Scanning (high switch, very short dwell)	8 (Large = 6, Small = 2)	2.902 (0.351)	0.075 (0.026)	1.091 (0.118)
*F*(3, 65)	–	–	51.35	73.61	38.34
*p*	–	–	< 0.001	<0.001	< 0.001
η^2^	–	–	0.703	0.773	0.639

Values are Mean (SD) of participant-level process-level exploration metrics computed from AOI sequences during the first 300 s. Switch Rate: higher values indicate faster scanning rhythm; Median State Duration: higher values indicate longer dwell/greater focal persistence; Transition Entropy: higher values indicate more diverse and less repetitive transition structure across AOIs.

At the metric level, Rapid–Scanning (Cluster 3) showed the highest Switch Rate and the shortest Median State Duration, consistent with fast scanning across AOIs. In contrast, Slow–Focal (Cluster 2) showed the lowest Switch Rate and the longest Median State Duration, consistent with sustained and focal exploration. Notably, Moderate–Diverse (Cluster 0) and Moderate–Repetitive (Cluster 1) exhibited comparable switching levels but diverged sharply in Transition Entropy (Cluster 0 > Cluster 1), indicating that even with similar switching frequency, some children transitioned across a broader set of AOIs whereas others cycled repeatedly among a narrower subset.

Cluster membership was strongly associated with classroom size, χ^2^(3) = 24.63, *p* < 0.001, indicating that exploration patterns were not randomly distributed across conditions. In the Large-room condition, children most frequently exhibited Moderate–Diverse exploration (Cluster 0; 41.7%), characterized by moderate switching combined with the highest transition entropy, suggesting flexible exploration across multiple AOIs rather than repeated cycling among a narrow subset. Moderate–Repetitive exploration (Cluster 1) accounted for the second-largest share in the large-room condition, whereas another conspicuous tendency in the large-room condition was Rapid–Scanning (Cluster 3; 16.7%), marked by the highest switch rate and shortest dwell durations, consistent with fast global scanning in a larger space.

In contrast, the Small-room condition was dominated by Moderate–Repetitive exploration (Cluster 1; 60.6%), which showed switching levels comparable to Cluster 0 but substantially lower entropy, indicating a more repetitive transition structure (i.e., frequent switching among a limited set of AOIs). The Small-room condition also showed a sizable Slow–Focal subgroup (Cluster 2; 33.3%), characterized by low switching, long dwell, and low entropy, consistent with sustained attention to fewer areas.

Overall, these patterns suggest that classroom scale shapes the temporal organization of gaze exploration: larger spaces more often elicited either diversified exploration at moderate switching rates or rapid scanning, whereas smaller spaces more frequently yielded repetitive switching within constrained AOI subsets or focal persistence.

#### 3.2.3 RQ4: do child characteristics predict process-level exploration cluster membership within each classroom-size condition?

To examine whether children’s individual characteristics were associated with process-level exploration profiles in the classroom-size comparison, we fitted a multinomial logistic regression model using maximum likelihood estimation. Cluster membership (*k* = 4) served as the outcome variable, with Cluster 0 (Moderate–Diverse) as the reference category. Predictors included child sex (boy = 0, girl = 1), age in months, and enrollment duration in months (Table 7).

**TABLE 7:** Multinomial logistic regression predicting classroom-size cluster membership.

Comparison (vs. Cluster 0)	Predictor	B (SE)	OR[95% CI]	*p*
Cluster 1 vs. 0	Sex	1.620 (0.812)	5.05 [1.03, 24.80]	0.046
	Age (in months)	−0.025 (0.097)	0.98 [0.81, 1.18]	0.796
	Enrollment (in months)	0.013 (0.031)	1.01 [0.95, 1.08]	0.675
Cluster 2 vs. 0	Sex	1.437 (0.701)	4.21 [1.06, 16.63]	0.041
	Age (in months)	−0.085 (0.085)	0.92 [0.78, 1.08]	0.314
	Enrollment (in months)	0.035 (0.027)	1.04 [0.98, 1.09]	0.194
Cluster 3 vs. 0	Sex	−0.050 (1.022)	0.95 [0.13, 7.04]	0.961
	Age (in months)	−0.141 (0.117)	0.87 [0.69, 1.09]	0.227
	Enrollment (in months)	0.036 (0.039)	1.04 [0.96, 1.12]	0.353

Model fit. Pseudo *R*^2^ = 0.0647; Log-likelihood = −81.41; LLR *p* = 0.2579. Outcome variable: cluster membership (*k* = 4). Reference category = Cluster 0 (Moderate–Diverse). OR = exp(B). *P*-values are reported descriptively; given the non-significant omnibus model, coefficient-level contrasts are interpreted as exploratory and hypothesis-generating.

The overall model did not significantly improve upon the intercept-only model (LLR *p* = 0.2579; Pseudo *R*^2^ = 0.0647), indicating limited evidence that these individual-level characteristics as a set predicted cluster assignment. At the coefficient level, child sex yielded two contrasts that reached nominal significance: higher values of the sex variable (i.e., girls in the present coding scheme) were associated with greater odds of membership in Cluster 1 (Moderate–Repetitive) relative to Cluster 0 (OR = 5.05, *p* = 0.046) and in Cluster 2 (Slow–Focal) relative to Cluster 0 (OR = 4.21, *p* = 0.041). Age and enrollment duration were not significant predictors in any comparison (all *p*s > 0.19), and no predictors reached significance for Cluster 3 (Rapid–Scanning).

Given the non-significant overall likelihood-ratio test, these coefficient-level contrasts should be interpreted as exploratory and hypothesis-generating rather than confirmatory. Although two sex-related contrasts reached nominal significance, they should not be taken as evidence of reliable sex differences. Accordingly, we refrain from drawing substantial inferences from these contrasts. Overall, the results suggest that, within this sample, dynamic exploration strategies in the classroom-size condition were more strongly structured by spatial scale (RQ3) than by measured child characteristics, with individual differences showing limited and non-robust predictive value.

## 4 Discussion

This study investigated preschoolers’ early visual exploration of immersive VR preschool classrooms using eye tracking, focusing on how architectural features shape where children look (AOI prioritization) and how they explore over time (transition dynamics). Across the first 5 min of exposure, we observed systematic differences in both gaze allocation and gaze-transition structure as a function of (a) window-view content (city vs. nature) and (b) classroom size (large vs. small). Importantly, transition-based clustering revealed qualitatively distinct exploration patterns that were strongly associated with environmental design features, suggesting that early “orientation” in a classroom is not only a matter of salience in isolated elements, but also of how children perceive the size of a space and repeatedly return to anchors and expand outward across activity areas.

### 4.1 Windows as distal anchors: content and strategic sampling

The window-view comparison indicates that preschoolers’ initial looking is sensitive to what is seen through openings. In the nature-view classroom, children devoted relatively more gaze time to window-related AOIs and showed stronger return-to-window tendencies, whereas in the city-view classroom the central window more strongly concentrated window-directed gaze. These differences suggest that “window effects” are not homogeneous: the same architectural feature can reorganize attention depending on the distal scene’s visual composition and informational structure.

Recent child scene-viewing evidence supports the plausibility of a content-driven mechanism. For example, children’s fixation behavior in streetscapes varies with scene properties such as greenery and visual richness; higher green-view ratio and greater vegetation variety have been discussed as factors associated with increased fixation durations, whereas monotonous gray, cement-like material scenes may dampen exploratory interest (Sheng et al., 2025). Translated to classroom windows, nature views may sustain attention (longer engagement) and invite repeated re-checking, potentially because they provide more perceptually rich and salient visual information (e.g., greater color/contrast variation and edge density) that can guide early fixation behavior in scene viewing (Henderson, 2003; Itti and Koch, 2000), while other views may pull attention differently (e.g., toward a single dominant aperture or toward indoor elements). Such content-driven attraction does not, however, by itself specify the function of later revisits within the scanpath: a window may first attract gaze because it is visually rich, yet subsequently be used as a stable reference during continued exploration.

Crucially, the observed temporal signature—returning to the window as exploration proceeds—aligns not only with the broader idea that openings can function as gaze anchors, but also with developmental theories of children’s spatial knowledge. In Siegel and White’s (1975) classic framework, children’s spatial representations progress from landmark knowledge to route knowledge and later to more integrated survey knowledge. From this perspective, repeated return to a stable distal cue can be read as an early, landmark-supported means of structuring a novel room before a richer route-like representation has been established. Empirical developmental work is consistent with this interpretation: children first acquire general landmark knowledge and only later become more able to select functionally useful landmarks and bind them to directional information (Nys et al., 2015), and even 5-year-olds improve route learning when their attention is explicitly drawn to on-route landmarks during initial exposure (Lingwood et al., 2015). Related work further suggests that landmark-based navigation is already measurable at ages 5–6, but that viewpoint-independent allocentric use of landmarks is still emerging and remains sensitive to cue type and task demands (van Hoogmoed et al., 2022). Taken together, this body of work makes it plausible that, once they have attracted attention, windows can serve as orientation-relevant reference structures during early classroom viewing.

This anchor-based interpretation also resonates with developmental work showing that improved spatial reasoning is not necessarily driven by more looking overall, but by more efficient allocation to diagnostically relevant cues. Hsieh (2025), for instance, reported that preschoolers’ gains in relational spatial reasoning were associated with faster access (shorter time-to-first-fixation) to target landmarks and reduced re-engagement with distracting cues, rather than increased total fixation time. This distinction is analytically useful for asking whether the repeated window revisits in the present study reflect primarily visual preference, a calibration-like orienting process, or both. Developmental reorientation research, together with the present transition results, suggests that a calibration-like process may have contributed to the observed revisit pattern, although it cannot be established conclusively here. For example, Lee et al. (2006) showed that a salient landmark can act as a direct cue to object location without necessarily serving as a directional signal for reorientation. Complementing this, Learmonth et al. (2002) showed that young children did not use an available landmark in the same way across spaces: landmark-guided reorientation was more evident in a larger enclosure than in a smaller one, implying that whether a visible cue functions as a usable orienting reference depends not only on its salience but also on the spatial scale in which it is encountered. Applied to the present study, a preference account would suggest that children looked back at the window because the view was visually rich or affectively appealing, whereas a calibration account would suggest that children intermittently revisited the same distal opening to re-establish orientation or a usable spatial reference while sampling other classroom areas.

These accounts are not mutually exclusive: visually rich content may have helped capture attention to the window in the first place, while subsequent revisits may have reflected reuse of that opening as a stable distal reference during ongoing exploration. Within this broader interpretation, a calibration-like contribution is especially plausible in the present paradigm because all children began from the same central seated position while facing the front wall and central window, making the window the most stable distal cue available at the outset of exploration. The transition results are likewise compatible with this reading: the return-anchored cluster was defined by higher return-to-window tendencies and more frequent same-window returns after short excursions, together with lower two-step expansion, rather than simply by increased overall looking at the window. Given that true landmark–direction binding and viewpoint-independent allocentric navigation are still developing at ages 5–6 (Nys et al., 2015; van Hoogmoed et al., 2022), repeated window revisits are best interpreted here as a developmentally plausible, landmark-supported pattern of spatial scaffolding during early orientation to an unfamiliar classroom; however, the present design does not allow the relative contributions of visual preference and calibration-like reuse to be decisively separated.

### 4.2 Room scale and depth structure: exploration rhythm, diversity, and distal bias

Classroom size was associated with systematic differences in early exploration. Beyond modest AOI-level differences, transition-based clustering revealed distinct dynamic profiles: large classrooms more often elicited high-diversity and/or rapid-scanning patterns, whereas small classrooms more often elicited slower focal viewing or repetitive cycles among fewer AOIs. A parsimonious interpretation is that larger spaces invite broader sampling to establish a global “scaffold” of layout, while smaller spaces facilitate more local exploitation—either because zones are less visually separable, or because nearby objects occupy a larger portion of the field of view.

Relevant adult VR findings suggest that spatial scale and depth cues can shift what becomes a reference structure during perception. In redirected-walking research, Kim et al. (2022) reported that gaze distributions in empty virtual rooms tended to align with a virtual-horizon reference across room sizes, whereas furnishing introduced objects that competed for attention and functioned as distractors. This supports a general mechanism whereby, when few mid-field anchors exist, observers rely on distal/structural cues (e.g., horizons or far boundaries), but when salient objects are present, they can capture gaze and reorganize perceptual sampling. As for the classroom-size condition in the current study, this implies that scale effects on exploration rhythm may partly reflect how strongly distal structural cues (front wall/window depth) compete with object-based anchors (e.g., book area/canopy).

More broadly, architectural proportion studies using eye tracking suggest that longitudinal depth can shape how quickly and how persistently observers engage with distal focal zones. For example, increasing nave length has been reported to shorten time-to-first-visit and increase fixation duration on the presbytery, consistent with stronger visual magnetism and less dynamically distributed scanning in longer-depth conditions (Rusnak et al., 2019). In combination with the well-documented central fixation bias in early scene viewing (Tatler, 2007), this suggests that spatial depth and frontal alignment can bias early viewing toward visually central and distal structures. In the present study, where all children began from the same central starting position while facing the front wall, this mechanism may help explain why attention was often drawn to visually central and frontally aligned regions during the earliest phase of classroom viewing. Relatedly, work using VR to study spatial updating in children highlights that self-motion and landmark cues can differentially support orientation, implying that early orientation from a fixed starting position may be especially shaped by the availability of distal visual references before locomotion begins (Barhorst-Cates et al., 2021).

### 4.3 Multiple anchors beyond windows: child-relevant micro-features and affordances

Windows were not the only candidate anchors in the scene. Across several conditions, the book/reading area captured substantial attention—especially when it contained visually distinctive, child-relevant micro-features (e.g., canopy/cushions). In the city-view condition, despite the presence of a large central window, the window appeared to function as a comparatively weaker anchor than in the nature-view condition, and the book area nonetheless accounted for the largest share of gaze. Similarly, in the large classroom condition, the book area received the highest proportion of visual attention. In contrast, in the small classroom condition—where the canopy and cushions were not present—the book area accounted for a comparatively smaller share of gaze. These patterns suggest that when a strong distal anchor is weakened (by view content, geometry, or relative prominence), internal “places” can serve as alternative anchors. One plausible mechanism is that bounded micro-spaces (e.g., a canopy-defined nook) increase perceptual coherence and “place-likeness,” thereby drawing gaze and organizing transitions. Another is that soft/comfort-associated furnishings may carry affective affordances that bias attention even without explicit task goals. At present, we cannot disentangle salience-driven (contrast/texture/shape), semantic (books/familiarity), and affective (coziness/safety) accounts; targeted manipulations of micro-features while holding room scale constant would clarify which mechanism dominates.

### 4.4 Individual differences: limited predictive signal in the current design

Age, sex, and enrollment duration provided limited explanatory power for cluster membership relative to the environmental manipulations. Within the constrained age range, early exploration strategies appeared more condition-sensitive than trait-sensitive. Still, exploratory contrasts in the classroom-size condition suggested that girls tended to show more repetitive and slower, focal exploration profiles than boys, although this pattern should be interpreted cautiously given the non-significant overall model fit. However, these results should not be taken to imply that individual differences are unimportant; future research with larger samples and more comprehensive child-level measures will be needed to more fully examine these effects.

### 4.5 Practical implications for early-childhood learning environments

The findings motivate several design hypotheses. First, window view content may influence whether windows operate as stable distal anchors during initial classroom exposure. If the design goal is to support calm orientation via repeated referencing to a stable distal cue, nature views may be more likely to support anchor-and-return dynamics, consistent with the broader notion that exterior connection and openness shape engagement and appraisal (Gao et al., 2025; Tural and Tural, 2024). Moreover, prior research has suggested that views containing natural elements can meaningfully alter experiences in learning settings, including perceived stress, attentional states, and classroom-related outcomes (Benfield et al., 2015; Kaplan, 1995; Lindemann-Matthies et al., 2021). From this perspective, the possibility that natural elements may capture preschoolers’ gaze during initial classroom exposure is encouraging. Importantly, this anchoring function may be relevant not only to comfort but also to how children rapidly structure a novel space: prior work suggests that preschoolers’ spatial reasoning benefits from more efficient visual strategies centered on quickly accessing relevant landmarks and minimizing revisits to distracting cues (Hsieh, 2025). From this perspective, a well-positioned distal anchor (e.g., a window) may support early “spatial scaffolding” during initial classroom exposure, provided it does not monopolize attention.

Second, the present results suggest that whether a window supports spatial scaffolding or instead produces anchor dominance depends not only on view content, but also on how the opening is positioned relative to nearby activity zones. In the current study, all children began from a common central position while facing the front wall, making visually central and frontally aligned distal structures especially likely to organize the first phase of looking. Under such conditions, a large frontal opening may function as an effective orienting reference, but it may also hold attention too strongly when the principal activity zone lies directly on the same visual axis. A more specific design implication, therefore, is not simply to reduce window salience, but to preserve a stable distal window that remains visible from common entry or initial-seating viewpoints while locating a primary activity zone laterally adjacent to, rather than directly aligned with, the strongest opening. This relational placement may allow the window to provide a reference for early orientation while increasing the likelihood of inward transitions to nearby classroom areas. The present data are consistent with this interpretation: in the nature-view condition, greater sampling of the small side windows co-occurred with greater attention to the adjacent Block and Dramatic Play areas, suggesting that openings can channel exploration toward nearby zones rather than only capturing gaze at the window itself. At the same time, the results also indicate that inward activity zones need their own counter-anchors. The Book Area drew substantial attention when it included visually distinctive child-relevant micro-features such as a canopy and cushions, but a smaller share when those features were absent. Accordingly, designers who wish to encourage faster inward engagement may need to consider window geometry (size, placement, multiplicity) and controllable modulation (e.g., shading or curtains) together with the placement of salient interior micro-features, especially when visually rich nature views may sustain repeated re-checking (Benfield et al., 2015; Hsieh, 2025; Lindemann-Matthies et al., 2021; Sheng et al., 2025; Tural and Tural, 2024).

Third, classroom scale appears to shape exploration rhythm. Larger spaces may invite rapid global sampling, while smaller spaces may support more focal engagement with fewer targets. However, adult findings also indicate that elongated depth can concentrate attention on distal focal structures (Rusnak et al., 2019), suggesting that designers should consider not only floor area but also depth structure and the placement of visually terminal anchors.

### 4.6 Limitations and future directions

Interpretation is bounded by several limitations. First, the study used VR rather than physical classrooms; VR environments may differ in depth cues, locomotion affordances, and device-specific display characteristics, potentially shaping perceptual and physiological responses (e.g., Cho et al., 2025). In addition, the present analyses were restricted to the initial seated phase (0–300 s), during which children viewed the classroom from a standardized central starting position. The findings therefore capture early visual orienting under fixed-start conditions rather than naturally occurring classroom entry and settling-in, which involve locomotion, social interaction, and evolving goals that may reorganize both AOI priorities and transition structure (Kattenbeck et al., 2025). In occupied classrooms, teachers and peers act as socially salient moving actors; faces, gestures, body orientation, and motion cues may compete with—or at times outweigh—static architectural features. Their presence could therefore fundamentally reorganize both the visual hierarchy itself (e.g., increasing prioritization of people or socially active zones) and the transition structure (e.g., more person-to-object and person-to-person shifts, and fewer repeated returns to distal architectural anchors). Accordingly, the present findings are best interpreted as a baseline characterization of architecture-driven early orienting under socially simplified conditions, rather than as a complete account of gaze behavior in occupied classrooms.

Second, in the classroom-size comparison, room scale could not be manipulated in complete isolation. The small room necessarily contained fewer furnishings, some activity areas occupied different positions relative to the common starting position and lateral visual field, and distinctive micro-features—most notably the canopy/cushion configuration in the book area—may have independently attracted gaze. Accordingly, some observed differences may reflect layout position, visibility, and local object salience in addition to room size *per se*. Furthermore, reducing room size geometrically increases the relative visual prominence of individual AOIs within the field of view, which may have additionally contributed to the greater gaze allocation observed in certain areas under the small-classroom condition. Likewise, in the window-view comparison, differences in daylight spill, luminance, or window-mullion reflection patterns between the city and nature scenes may also have contributed to window-related gaze (e.g., Amundadottir et al., 2017). Thus, part of the observed AOI differences may reflect recurrent early-stage scanning tendencies under the common starting orientation, interacting with positional visibility, rather than differences in window-view content and room size alone.

Methodologically, although the headset’s nominal eye-tracking rate was 120 Hz, gaze was synchronized and logged at 50 Hz for analysis. This approach was driven by the necessity to map eye tracking and behavior data onto a common time axis. Recording gaze at 120 Hz would have generated frames lacking corresponding behavioral data due to the 50 Hz system update cycle. Additionally, logging data at the maximum frequency could have increased system load and introduced latency, potentially degrading the user experience. Therefore, 50 Hz logging was considered an optimal approach to ensure synchronization and system stability. However, very brief fixations or other fine grained oculomotor events might have been underestimated relative to higher frequency logging. Specifically, inter-AOI transition counts may be slightly underestimated and estimated dwell durations may be marginally inflated. Future studies employing continuous high-frequency gaze logging throughout would provide additional precision for analyses of brief orienting responses.

Additionally, AOI assignment was based on per-object raycasting, applying collision detection to the precise mesh geometry of each virtual object rather than to approximated bounding boxes. For partially open furnishings, rays can pass through visible apertures and register collisions with objects behind them, thereby minimizing gaze occlusion errors. Furthermore, because the final analyses were conducted at the level of activity areas rather than individual objects, partial occlusions among objects belonging to the same activity area were unlikely to meaningfully alter the final AOI label. Nevertheless, in the large-classroom condition, the shelving unit between the Book Area and the Music Area may have partially occluded the Music Area from certain viewpoints, potentially contributing to the relatively lower gaze proportion recorded for that area.

Next, because the VR session was embedded in a broader laboratory protocol that also included saliva sampling and computer-based tasks during the same visit prior to VR exposure, which took approximately 15 min, fatigue or cognitive-load carryover effects cannot be fully ruled out, although all participants underwent the same overall procedure.

Finally, the manipulations were analyzed as separate between-subject contrasts rather than a full factorial design, so interactions between view content and room size cannot be inferred. Clustering results should therefore be interpreted as exploratory, and replication together with validation against downstream outcomes (e.g., spatial memory, classroom engagement, wayfinding, and affect) remains necessary. Supplementary bootstrap analyses further indicated that, under the selected four-cluster solution for the classroom-size comparison, a rapid-scanning–like profile repeatedly re-emerged across resamples. We therefore interpret Rapid-Scanning as a reproducible but low-frequency exploration profile within the present data. At the same time, because its recovered size varied across bootstrap resamples, the exact boundary and broader generalizability of this profile should be interpreted cautiously and verified in larger samples. Future studies should combine free exploration with goal-directed tasks and incorporate physiological or behavioral measures to test whether anchor-and-return dynamics predict functional adaptation, and whether similar gaze-allocation patterns re-emerge in larger samples.

## 5 Conclusion

Preschoolers’ early visual exploration of VR preschool classrooms appears to be systematically shaped by architectural design features. Window-view content was associated with both AOI-level allocation and distinct scanpath organizations, including return-to-window anchoring versus more expansion-oriented exploration. Classroom size similarly related to differences in both where children looked and how they transitioned among zones, indicating that room scale shapes the rhythm and diversity of early orientation. Overall, the findings support VR eye tracking with transition-based scanpath metrics as a promising approach for evidence-informed design of learning environments for young children, and they highlight the value of conceptualizing windows not merely as attention attractors but as potential reference anchors that structure how young children visually organize a novel classroom during initial exposure. In addition, room scale may be associated with differences in exploration tempo, potentially relating to faster scanning and transition patterns in larger spaces versus more repetitive and focal viewing in smaller spaces, although these dynamics warrant further empirical clarification.

## Data Availability

The raw data supporting the conclusions of this article will be made available by the authors, without undue reservation.
